# Ecological Effects of Heavy Metal Pollution on Soil Microbial Community Structure and Diversity on Both Sides of a River around a Mining Area

**DOI:** 10.3390/ijerph17165680

**Published:** 2020-08-06

**Authors:** Xingqing Zhao, Jian Huang, Xuyan Zhu, Jinchun Chai, Xiaoli Ji

**Affiliations:** 1 School of Environmental and Safety Engineering, Changzhou University, Changzhou 213164, China; huangjian08170@163.com (J.H.); xuyan_zhu@foxmail.com (X.Z.); 2 Department of Civil Engineering and Architecture, Saga University, Saga 8408502, Japan; chai@cc.saga-u.ac.jp; 3 School of Economics, Changzhou University, Changzhou 213164, China

**Keywords:** Tongling mining area, river banks, heavy metal pollution, microbial community structure, Illumina MiSeq

## Abstract

The objectives of this study were to understand the characteristics of heavy metal pollution caused by mining activities on the two sides of the Shun’an river and the response of soil microorganisms to the habitats by different contamination levels and vegetation. This paper selected soil samples from the banks of the Shun’an River near the Shizishan mining area, which is at the left of the river, in Tongling, Anhui Province, China. Using Illumina MiSeq 2500 technology, we analyzed the relationship between environmental factors and microbial communities. As the distance from the mining area increased, the heavy metal comprehensive pollution and potential risk value decreased. Additionally, the pollution severity and risk value of the left bank, where the mining area lies, were generally higher than those of the right bank. Because the symmetric sampling points on both banks of the river had similar planting types, their environmental factors and microbial community structure were similar and clustered. However, under different vegetation, the paddy soils tended to have a higher nutrient content and community richness and diversity than the vegetable fields or the abandoned land. It was found that soil microbial communities in this area were mostly affected by pH and Nemerow pollution index (P*_N_*). The pH significantly affected the abundance and structure of most microorganisms. In addition, Proteobacteria, Acidobacteria, and Bacteroidetes had significant tolerance to Zn, Pb, and Cd. By exploring the potential use of these tolerant microorganisms, we seek to provide strains and the theoretical basis for the bioremediation of areas contaminated by heavy metal.

## 1. Introduction

The mining of mines has a devastating impact on the global environment [1,2]. As a large number of mines are exploited, a large amount of acid mine drainage (AMD) and waste slag are released [3], which causes serious pollution of the water [4] and soil around the mines (such as paddy fields, vegetable fields, and abandoned land). As a result, the contaminated area has an impact on the quality of agricultural products, causing serious economic losses, entering the human body through the food chain, and endangering human health and life [5,6].

Soil microbes are involved in almost all biochemical processes in the soil ecosystem, and they are responsible for regulating soil ecosystems, maintaining the earth’s material circulation, and buffering and purifying polluted soil [7]. Soil microbes are more sensitive to environmental stress than small animals and plants in the same habitat, so soil microbial activity is considered a sensitive indicator of soil ecosystems [8].

Our previous study found that soil physicochemical properties and heavy metals significantly affected the microbial compositions, and the microbial community structure was significantly variable depending on different vegetation types and depth layers in mines [9]. Tischer et al. [10] found that the structure of the soil microbial community had good adaptability to the environmental change of vegetation through the investigation of natural forests, shrub lands, young grazing lands, old grazing lands, reconstructed grazing lands, and abandoned grazing lands. Numerous studies have shown that microorganisms are particularly sensitive to changes in soil’s physicochemical properties (such as temperature, moisture content (MC), pH, nutrient availability, or clay content) and pollution (e.g., heavy metals) [11,12]. These factors directly control the abundance and activity of microorganisms, resulting in changes in microbial community structure and diversity [13,14]. Around a mining area, environmental gradients may be decisive factors for microbial diversity, abundance, and composition, such as AMD, heavy metal pollution, pH, and other environmental gradients [15]. In general, excessive traces of metals can cause toxicity, and contaminated soil acts as a sieve to reduce microbial diversity while enriching tolerant species [16]. These enriched tolerant species are due to their active use of various physicochemical processes to immobilize the heavy metals in the soil, such as adsorption, precipitation, and binding, which fixes the heavy metals in the soil, thereby reducing environmental toxicity to allow species to adapt to the new environment [17].

For a long time, high loads of heavy metal ions produced in a mining area have entered the surrounding soil and rivers through processes, such as the leaching of tailings and AMD discharge, resulting in widespread concern about pollution of the surrounding soil environment [18]. Some researchers have studied the microbial communities around mines [18,19]. By studying the Picher mine that is now closed and the different cross-sections extending 8.05 km around the mine, Beattie et al. [20] found an uneven distribution of heavy metal pollution, but the microbial community structure changed with increasing metal concentration, and the total bacterial density decreased. Fan et al. [21] studied soil microbial distribution in four sampling areas (influent, upstream, downstream, and effluent) of mine drainage and found that microbial communities moved along the direction of mine drainage, and heavy metals were the key determinants of microbial communities. In addition, the pollution status of different land-use types in soil around mining areas has been investigated, especially in farmland areas [22,23]. Asakawa and Kimura [24] found that the various microbial communities of different habitats in a paddy field system were completely different in terms of diversity and stability. However, the response of microbial communities to metal contamination and soil properties under different land vegetation still have not been well-studied. Therefore, a wealth of knowledge regarding the changes of microbial community structure related to land vegetation is of great significance for land cultivation, management, and restoration in mine areas.

Here, we used Illumina MiSeq 2500 sequencing technology to characterize the distribution of the soil microbial communities under different plant types on both sides of the Shun’an River around the Shizishan Mining Area in Tongling City. The main purposes of the study were as follows: (1) to characterize the diffusion of soil pollution on both sides of the river; (2) to provide new insight into the abundance and diversity of soil microbial communities under different planting types in farmland around the mining area; and (3) to quantify the relationship between environmental parameters and microbial communities. This research will help us to understand how the physicochemical properties and heavy metals of soils related with different land vegetation affect microbial assemblages.

## 2. Materials and Methods

### 2.1. Field Description and Sample Collection

Shizishan Mine is located in Tongling City, Anhui Province, China, and is the main mining area for Cu, Ag, Fe, and other metals. It is located on the left bank of the Shun’an River, and the river passes around the mine and then flows from west to east into the Yangtze River. The annual average rainfall is 1346 mm, and the annual average temperature is 16.2 °C. Due to a large number of wastes that are generated during the mining, processing, and transportation, the pollutants from these wastes diffuse to the surrounding through precipitation or acidification, polluting the soil and water nearby. Previous investigations in the Tongling mining area have shown that the soils and surface waters around the mining area were contaminated to varying degrees by toxic metals [25]. Therefore, there are serious environmental problems in the soil on both sides of the river around the mining area [26], and the research on the soil around the river in the mining area will be representative.

**Figure 1:**
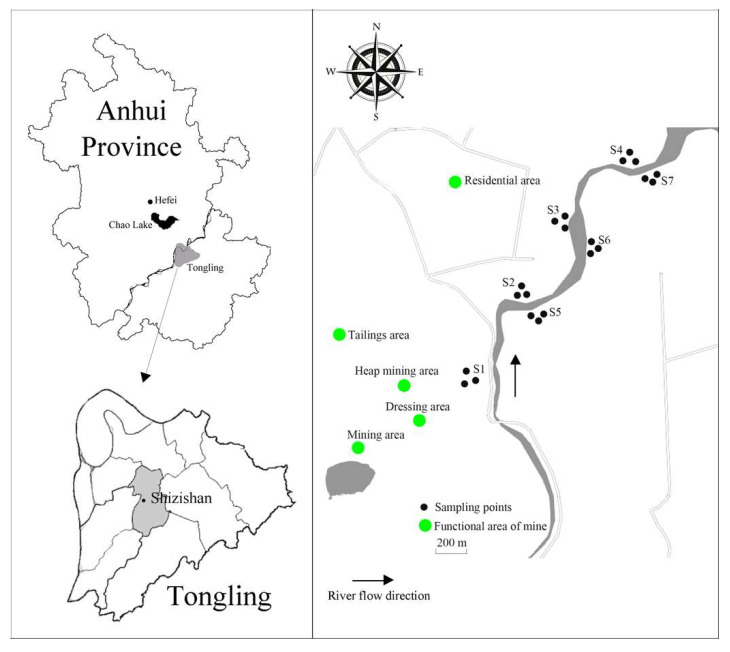
Map of Anhui Tongling Mining area and sampling points. Note: Abandoned land (S1) (S1-1, S1-2, S1-3), wasteland near the mine; vegetable field S2 (S2-1, S2-2, S2-3), S5 (S5-1, S5-2, S5-3), near the residential area and corn belt; paddy field S3 (S3-1, S3-2, S3-3) and S4 (S4-1, S4-2, S4-3), S6 (S6-1, S6-2, S6-3), and S7 (S7-1, S7-2, S7-3).

In May 2018, taking the Shizishan mining area as the center, samples were collected from an area extending 500 m outward from each side of the Shun’an River (Figure 1). According to different vegetation (such as farmland, vegetable fields, and abandoned land), samples were collected from four areas (S1, S2, S3, S4) on the left bank of the river and from three areas (S5, S6, S7) on the right bank of the river. S7 2 km away from the mine with relative low level of disturbance was selected as the reference soil. In each area, three soil samples were randomly collected with a drill bit (diameter 6 cm) from the 0–20 cm layer using the plum blossom point method, and were mixed to form a composite sample (e.g., S1-1). Three composite samples (S1-1, S1-2, S1-3) were collected from each area and were packed into separate sterile plastic bags and marked. Twenty-one composite fresh samples from seven sampling areas were immediately placed on ice and shipped to the laboratory. The soil samples from the seven sampling areas on both sides of the river around the mine were divided into three different types of soil as follows: the left bank of the river, that is, the abandoned land area S1 (S1-1, S1-2, S1-3) near the mine quarry, the vegetable field area S2 (S2-1, S2-2, S2-3) near the residential area, the paddy field area S3 (S3-1, S3-2, S3-3) and S4 (S4-1, S4-2, S4-3), and the right bank of the river, that is, the vegetable field area S5 (S5-1, S5-2, S5-3), and the paddy field area S6 (S6-1, S6-2, S6-3) and S7 (S7-1, S7-2, S7-3). All soil samples were homogenized through 2 mm nylon mesh to remove stones and plant roots, and were divided into two portions and placed into sterile self-sealing sample bags. One sample was stored in a refrigerator at 4 °C for soil physical and chemical analyses. The other sample was uniformly mixed (three fresh soil samples from the same area) to obtain seven mixed samples (S1, S2, S3, S4, S5, S6, S7), followed by storage in a −80 °C refrigerator for molecular analysis.

### 2.2. Soil Analysis and Physicochemical Determination

The moisture content (MC) of the soil was determined by drying to a constant weight at 105 °C ± 2 °C in a drying oven followed by weighing [27]. The pH was measured using a pH meter (PHS-3C, accuracy: ±0.01 pH, Shanghai INESA Instrument Co., Ltd., Shanghai, China) in 1:2.5 (soil/water) H_2_O suspensions after 1 h of shaking (180 rpm). The total organic carbon (TOC) of the soil was investigated by the K_2_Cr_2_O_7_ oxidation-external heating method [28]. Total nitrogen (TN) and total phosphorus (TP) of the soil were determined by the alkaline potassium persulfate digestion UV spectrophotometric method and the ammonium molybdate spectrophotometric (UV-2550, Shimadzu, Japan, measuring wavelength range: 190–900nm) method [29], respectively. Available potassium (AK) and total heavy metals (Cu, Zn, Pb, Cd, and Ni) were measured by a novAA 300FL flame atomic adsorption spectrophotometer (Jena Germany analytical instruments Co., Ltd.). Nemerow pollution index (P*_N_*) was calculated to evaluate the overall pollution of heavy metals in mine soil [30], and then, the potential ecological risk index (RI) was applied to assess the sensitivity of the environment to heavy metal pollution [31].

(1) $$C_{f}^{i}=\frac{C_{s}^{i}}{C_{n}^{i}}$$

(2) $$E_{r}^{i}=T_{r}^{i}\times C_{f}^{i}$$

(3) $$RI=\overset{n}{\underset{i=1}{\sum }}E_{r}^{i}=\overset{n}{\underset{i=1}{\sum }}T_{r}^{i}\times C_{f}^{i}=\overset{n}{\underset{i=1}{\sum }}T_{r}^{i}\times \frac{C_{s}^{i}}{C_{n}^{i}}$$

(4) $$P_{N}=\sqrt{\frac{{\left(C_{f}^{i}\right)}_{\max}^{2}+{\left(C_{f}^{i}\right)}_{\mathrm{ave}}^{2}}{2}}$$

Note: RI is the potential ecological risk index; PN is Nemerow comprehensive pollution index. $E_{r}^{i}$ is the environmental risk index of heavy metals (i); $T_{r}^{i}$ is the “toxic response” for the given substance; $C_{s}^{i}$ is the measured value of pollutant i content, mg/kg; $C_{n}^{i}$ is the background values of pollutant i content, mg/kg.

### 2.3. DNA Extraction, PCR Amplification and Illumina MiSeq Sequencing of the Soil

The DNA of samples was extracted using the MOBIO PowerSoil^®^ DNA Isolation Kit. The genomic DNA was amplified by PCR using the 16S rRNA gene V4 region primers (515F and 806R), barcode-specific primers, and Ta KaRa Premix Taq^®^ Version 2.0 [32]. The PCR conditions were as follows: denaturation at 94°C for 5 min, followed by 30 cycles (94 °C 30 s, 52 °C for 30 s, 72 °C for 45 s), and finally, an extension at 72°C for 10 min. The amplified product was verified by 1.0% agarose gel electrophoresis. The PCR (polymerase chain reaction) amplification product was further purified using a purification kit, and the PCR mixed product was recovered using an EZNA Gel Extraction Kit (Omega, Norcross, Georgia, USA). Finally, the Illumina MiSeq2500 (Guangzhou Magigene Biotechnology Co. Ltd., Guangzhou, China) platform was used for sequencing and microbial community analysis. The bacterial sequencing data were uploaded into the Sequence Read Archive (SRA) of NCBI (National Center for Biotechnology Information), and can be accessed through accession number PRJNA642756.

### 2.4. Statistics and Data Analysis

Data were entered in Microsoft Excel 2013 (Microsoft Corporation, Redmond, America). The low-quality reads in the original data were removed by Mothur software (V1.35.1, https://www.mothur.org/, Dr. Patrick Schloss and his resarch, Department of Microbiology and Immunology, The University of Michigan, America,), and the clean tags from different samples were determined separately from the row tag. Uchime software was used to remove the chimeras formed during the PCR, and the high-quality sequences obtained were clustered using the Usearch method [33]. The 97% similarity of the 16S rRNA gene sequence was used as a criterion for the classification of the operational taxonomic units (OTUs). The Chao1, Observed species, Simpson, Shannon diversity indices, and Principal Coordinate Analysis (PCoA) were calculated using the QIIME platform (http://qiime.org/), and the RDP classifier identified the microbial taxonomic status of the OTU representative sequence. Redundancy analysis (RDA) was a multivariate “direct” gradient analysis method. The significance of the variables was tested and automatically selected using Monte Carlo permutations [34]. Analysis of variance (ANOVA) and Spearman correlation analysis were performed using SPSS Statistics 22.0 (Statistical Graphics Crop, USA). The one-way ANOVA test was used to identify the homogeneity of the variance, and multiple comparisons were made by using the Dunett method of LSD. Significant effects were conducted using the Kolmogorov-Smirnov test, with significant differences identified where *p* < 0.05 [35]. Pearson correlation analysis was used to assess the relationship between physicochemical parameters [36]. RDA, VPA, Heatmap, and other graphs were analyzed using R software (V2.15.3) [33].

## 3. Results

### 3.1. Soil Physical and Chemical Properties and Heavy Metal Pollution

Table S1 shows the results of physical and chemical property detection and pollution characteristic evaluation of soil samples on both sides of the river in the mining area. Except for the S2 sampling point on the left bank of the river (weak alkaline PH = 7.79) and the S5 sampling point (neutral, pH = 7.02), other sampling points (S1, S3, S4, S6, S7) were weakly acidic (6.11–6.33). Soil nutrients (TOC, TN, TP, and AK) differed significantly between the abandoned land (S1) and other points, and the nutrients in the vegetable fields (S2) and paddy fields were significantly higher than those in the abandoned land. The TOC (3.69 g/kg), TN (977.46 mg/kg), and AK (53.45 mg/kg) of the abandoned land (S1) were significantly lower than the TOC (8.9–16.12 g/kg), TN (1190.94–2455.69 mg/kg), and AK (95.78–168.8 mg/kg) contents of the planted soil (S2, S3, S4, S5, S6, and S7). In contrast, the TP of S1 (801.61 mg/kg) was significantly higher than the TP content of S2–S7 (257.92–485.17 mg/kg). In addition, the soil moisture content was significantly higher in the paddy fields (S3, S4, S6, S7) than in the abandoned land (S1) and vegetable fields (S2, S5).

It can be seen from Table S1 that the soil on both sides of the Shun’an River was influenced to different degrees due to the diffusion of sewage from mining and river water irrigation. The background soil value of Tongling was used as a reference to analyze the heavy metal pollution in the soil samples [37]. The average value of heavy metals in most samples exceeded the background value, and the difference was significant. Cu and Cd seriously exceeded the background value, and the excess reached 5.62 times and 8.4 times of the background value, respectively. With respect to Pb and Zn, the average value of the soil was reached by 1.72 times and 2.41 times of the background value, respectively. The minimum level of Ni pollution was basically maintained at 0.68–2.05 times. The content of Zn in S3, S6, and S7, the Pb content in S5, S6, and S7 on the right bank, and the Ni content in S7 did not exceed the local soil background value.

According to the P*_N_* and RI index, the soil heavy metal pollution degree of different sampling points was ranked as S1 (13.18) > S2 (6.99) > S3 (6.20) > S5 (6.17) > S4 (5.51) > S6 (5.24) > S7 (3.35) according to P*_N_* index, whereas the potential risk index RI was ranked as S1 (609) > S2 (325) > S3 (280) > S4 (251) > S5 (233) > S6 (229) > S7 (149). S7 was the reference soil, and thereby had the lowest ecological hazard of all the detected heavy metals. It can also be seen that the values of *P_N_* and RI of the left bank and the residential area were higher than those of the right bank, and the comprehensive pollution risk value decreased with distance from the mine and downstream along the river.

### 3.2. Microbial Community Abundance and Structural Diversity

Using the Illumina MiSeq 2500 sequencing technology, the total bacteria and archaea of the seven soil samples were obtained after filtering the low-quality data, and the high-quality sequence numbers were 249,295 and 15,353, respectively. Cluster selection based on sequence similarities higher than 97% revealed 20,794 and 511 OTUs. Table S2 shows that the paddy field had more OTUs than the abandoned land and the vegetable land, that is, S7 > S6 > S3 > S4 > S5 > S1 > S2. The richness of species from the Chao1 index and the observed species showed a similar ranking. In addition, the biodiversity indicated by the Shannon and Simpson indices shows that the diversity of bacteria in the S2 region was relatively low, while the diversity of archaea was relatively high. Moreover, we found that the homogeneity of the bacteria across the seven sampling points was relatively high, and the uniformity of the archaea was poor, especially in the S5 region (Simpson’s index: 0.26).

**Figure 2:**
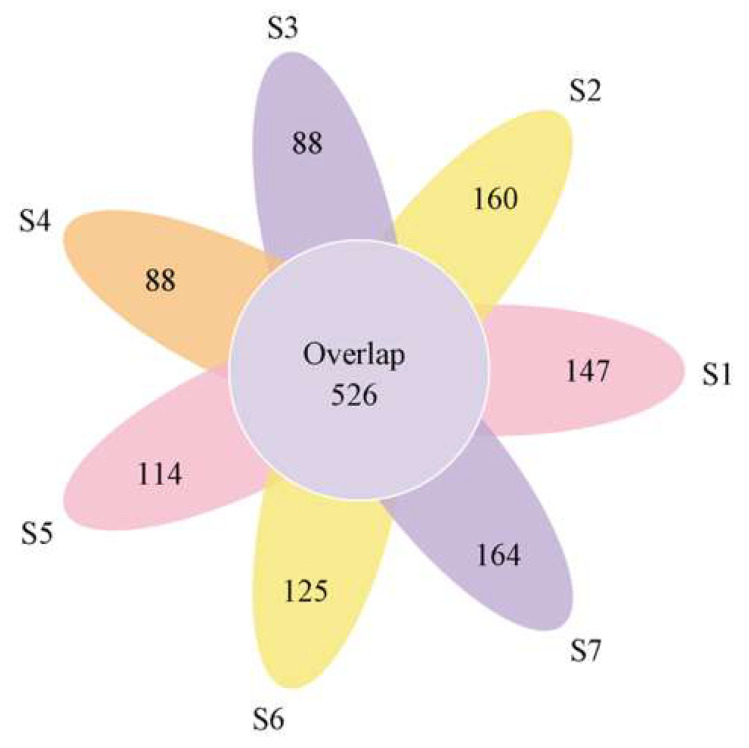
Venn diagram displaying the OTU (operational taxonomic unit) richness distribution in seven regions.

The dilution curve tended to flatten but did not reach saturation (Figure S1), indicating that the sequencing depth was sufficient to capture most of the microbial diversity in the soil. It can be seen that the area with less pollution had a relatively high level of diversity, while the area with a higher degree of pollution had a lower level of diversity. The Venn diagram (Figure 2) evaluated and categorized the OTUs of the seven sampling points and showed only 595 (2.8%) of the total OTUs, indicating that the microbial community differences among these seven regions were significant. The soils with similar land types had more common OTUs (Figures S2 and S3), which indicates that the soil microbes of the same soil planting type had high similarities, but each sampling area had its own unique microorganisms.

**Figure 3:**
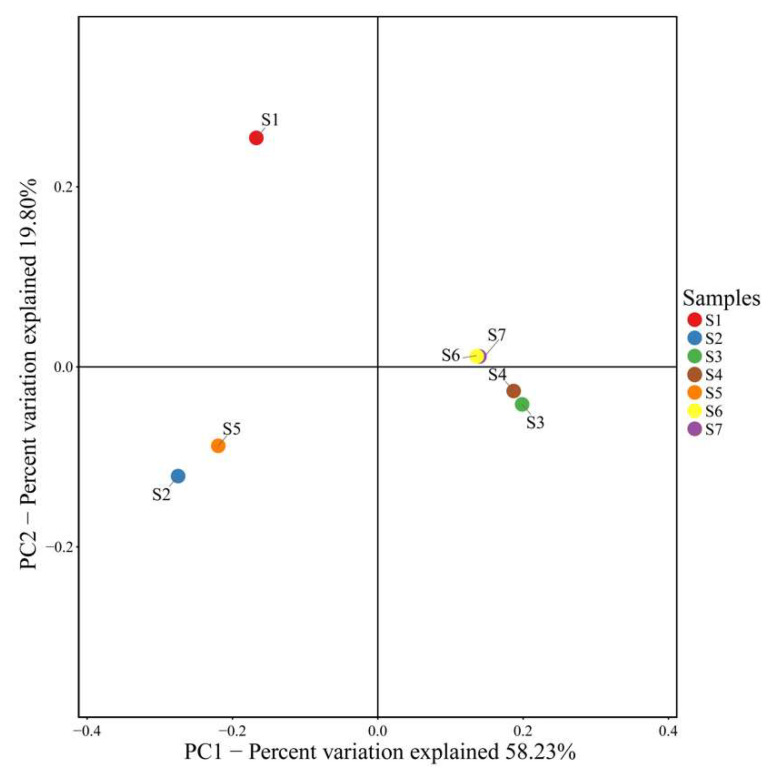
Principal coordinate analysis (PCoA) of sampling points and related environmental variables in the Tongling Mining area.

Principal Coordinate Analysis (PCoA) was used to further evaluate the similarity of species composition and the combined effects of environmental factors on the β-diversity of microbial communities in the seven different sampling areas (Figure 3). Nearly 77.23% of the difference in microbial community composition was explained (the first- and second-dimension interpretations were 58.69% and 18.54%, respectively) by the existing factors, indicating that the selected research factors resulted in significant differences in microbial community distribution and diversity. The PCoA results showed that the microbial communities of the same soil planting type from both sides of the river were similar and clustered (paddy fields, vegetable fields). This result is the same as the similarity measure metric heat map (Figure S4), that is, the S2 and S5 clusters were similar to the S1 cluster; the S3 and S4 clusters were similar, and the S6 and S7 clusters were similar.

**Figure 4:**
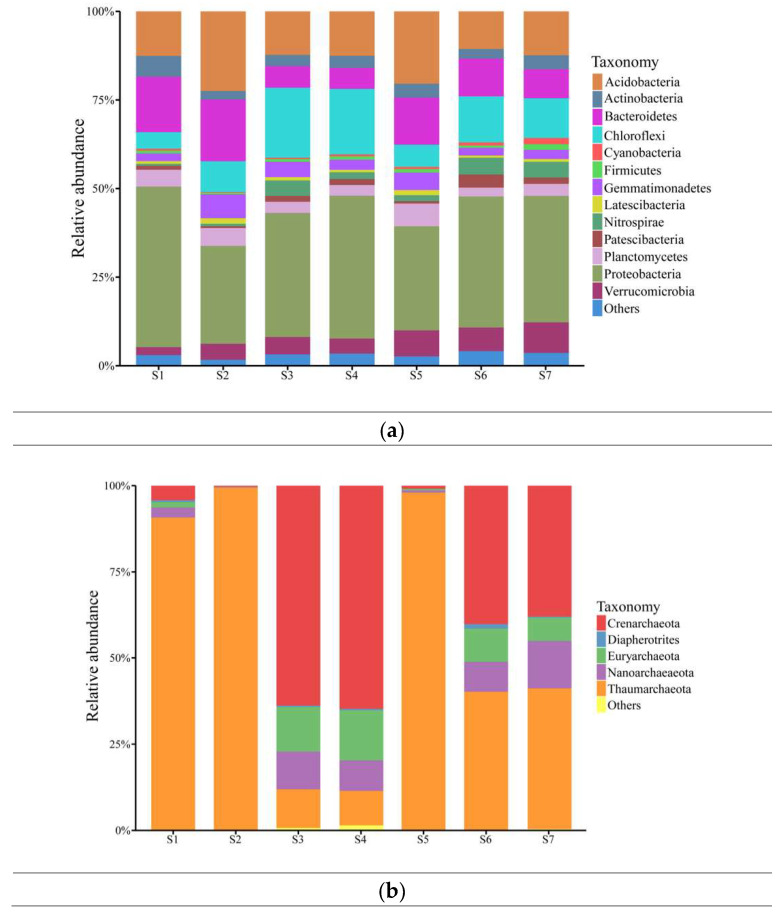
Relative abundance of microorganisms at the phylum level in soil samples from mining areas. (**a**) Percentage of bacterial richness, (**b**) percentage of archaea richness.

The soil microbial community was mainly composed of bacteria, accounting for 97.6% of the 16S rRNA sequence, while the archaea only accounted for 2.4% of the sequence. Specifically, the microbial community detected a total of 13 bacteria and five archaea at the phylum level (0.01) (Figure 4a,b). There were five dominant bacteria, Proteobacteria (34.91%), Acidobacteria (14.40%), Chloroflexi (11.44%), Bacteroidetes (10.77%), and Verrucomicrobia (5.36%) (relative richness > 5%) that accounted for 76.87% of the total number of bacteria. Some other bacteria were present in low numbers but were still detected in most samples, including Planctomycetes (3.95%), Gemmatimonadetes (3.59%), Actinobacteria (3.54%), Nitrospirae (2.57%), and Patescibacteria (1.55%). In addition, more than half of the archaeal sequences belonged to Thaumarchaeota (55.78% of all archaeal sequences), followed by Crenarchaeota (30.36% of all archaeal sequences), which accounted for 1.34% and 0.73% of the total microbial community, respectively. A total of 17 species of microorganisms were detected at the class level (Figure S5), and the dominant bacteria (relative richness >5%) were Gammaproteobacteria (17.99%), Deltaproteobacteria (9.79%), Bacteroidia (9.66%), Anaerolineae (9.40%), Alphaproteobacteria (7.09%), and Verrucomicrobiae (5.35%).

From the classification results of the microbial species at the phylum and class levels (Figure 4, Figure S5), it is known that the community in all soil samples had similar compositions of predominant bacteria, regardless of the contamination levels and vegetation. Nevertheless, heavy metal contents and vegetation affected their relative abundance, and the impacts varied among the taxa. The most dominant Proteobacteria accounted for the highest proportion of microbial communities (45.33%) in the abandoned soil, followed by the paddy soil (S3, S4, S6, S7) on both sides of the river, accounting for 35.02–40.31%, while the lowest proportion was in vegetable soil (S2, S5; 27.60% and 29.36%, respectively). Acidobacteria, Bacteroidetes, and Planctomycetes dominated in the abandoned land and vegetable fields. Conversely, Chloroflexi dominated in paddy soils on both sides of the river. In addition, Thaumarchaeota was also dominant in S1, S2, and S5, accounting for 90.77%, 97.98%, and 99.39%, respectively, of the archaeal community in the region. Crenarchaeota dominated in S3 and S7 (63.73%–64.76%). At the same time, the two archaea accounted for nearly 40% of the archaeal community in the S4 and S6 regions.

### 3.3. Relationship between Microbial Community Structure and Abundance and Environmental Factors

**Figure 5:**
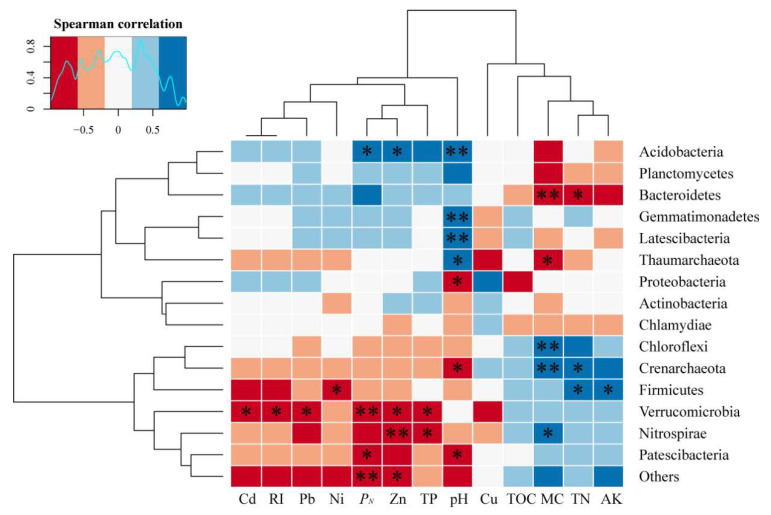
Heatmap of Spearman’s rank correlation coefficients combined with a cluster analysis between the different environmental factors and the relative abundances of microbial phylum. Note: Soil organic matter (SOM); Available nitrogen (AN); Available phosphorus (AP); Available potassium (AK); Total nitrogen (TN); Moisture content (MC). ** indicates an extremely significant correlation (*p* < 0.01), and * indicates a significant correlation (*p* < 0.05).

Correlation heatmaps (Figure 5 and Figure S6) were obtained by analyzing the correlation between soil physicochemical properties and the relative abundance of microorganisms (i.e., abundance > 1%). It was found that the pH was not related to Verrucomicrobia, but to other microbial phylum. In particular, it was significantly negatively correlated with Acidobacteria, Gemmatimonadetes, and Latescibacteria (*p* <0.01) and significantly negatively correlated with Proteobacteria and Patescibacteria (*p* <0.05). Proteobacteria, Acidobacteria, and Bacteroidetes were positively correlated with heavy metals and the comprehensive pollution index, and Bacteroidetes was significantly negatively correlated with MC and nutrients. Chloroflexi (11.44%) and Verrucomicrobia (5.36%) differed from the above dominant bacteria. Verrucomicrobia was significantly negatively correlated with Cd, Pb, Zn, *P_N_*, and RI and was positively correlated with nutrients.

The dominant class Gammaproteobacteria (17.99%), belonging to the Proteobacteria phylum, showed the same tendency as its phylum, while the Deltaproteobacteria (9.79%) and the Gammaproteobacteria had the same negative correlation with pH, and the other correlations were completely opposite to the Gammaproteobacteria, that is, Deltaproteobacteria were negatively correlated with pollution, such as heavy metals, and significantly positively correlated with MC and nutrients. Subgroup 6 and Blastocatellia [Subgroup 4] had the same correlation with Acidobacteria at the phylum level, while Acidobacteria was completely opposite to Acidobacteria, which was significantly negatively correlated with Zn, Pb, TP, *P_N_*, and pH. In addition, the class level of Anaerolineae and Bacteroidia were a completely consistent correlation with the phylum level of Chloroflexi and Bacteroidetes, respectively.

A study on archaea found that Thaumarchaeota showed a significant positive correlation with pH preference, a significant negative correlation with MC, and a negative correlation or no significant relationship with heavy metal pollution and nutrients. Crenarchaeota prefer a paddy field with high MC and high nutrient content, showing a significant positive correlation, but a significant negative correlation with pH and heavy metal pollution.

**Figure 6:**
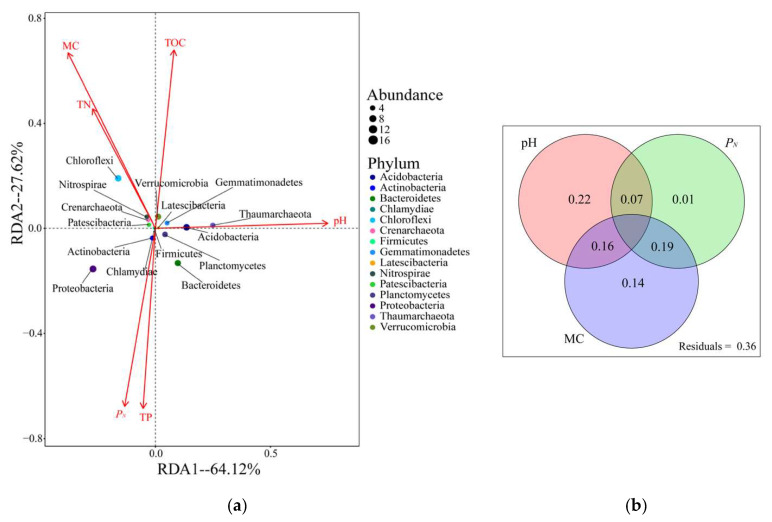
(**a**) Redundancy analysis (RDA) used to describe the relationship between microbes and environmental factors; (**b**) variance partitioning analysis (VPA) used to quantify the impact of environmental factors around the mining area on microbial communities.

The Monte Carlo test was used to analyze the relationships among environmental factors, communities, and distributions. We found six important environmental factors that affected the distribution of samples and microbial communities, namely, pH, MC, TP, TN, TOC, and *P_N_*. Furthermore, an RDA map (Figure 6a) and VPA map (Figure 6b) obtained by quantifying the environmental variables were obtained. The two most important axes accounted for 91.74% of the soil microbial community distribution and composition; RDA1 and RDA2 accounted for 64.12% and 27.62%, respectively. pH had the greatest impact on all environmental factors due to its smallest angle to the RDA1 value and the longest projection to the critical axis RDA1. MC had a certain influence on both axes. This result can also be confirmed in the VPA chart, that is, soil pH, MC, and *P_N_* contributed the most to the microbial community distribution, accounting for 64% of the total interpretation, and the remaining environmental interpretation was 36%.

## 4. Discussion

### 4.1. Physicochemical Properties of Soil and Characteristics of Heavy Metal Pollution

In recent years, soils near mining areas have been contaminated with heavy metals, which has long been considered a serious environmental problem [38]. We analyzed seven sampling areas distributed along the lower reaches of the Shun’an River around the Shizishan mining area in Tongling. The study found that S1 was the most polluted across all sampling locations (*P_N_* = 13.18 ± 1, heavy pollution; RI = 609.24, very strong ecological hazard) (Table S1). In the present study, abandoned land is located around the mining area, and the heavy metals that leach from these areas flow as surface runoff to the abandoned land, causing this area to be the most seriously polluted by heavy metals. This result not only shows that the closer to the mine area, the more serious the heavy metal pollution, but also indicates the important reason why that the heavy metal pollution on the left bank of the river was higher than that on the right bank is that the mining area lies on the left of the river. In addition, the pollution in the study area may also originate from heavy metal-containing domestic waste discarded by residents, and heavy metals flow to downstream fields during and after rainfall [22]. Fertilizer is applied to farmland over a long-term basis, and some studies have indicated that the applied fertilizer contains heavy metals (such as Cu, Zn, and Cd) [39]. On the whole, Cd pollution in this area is generally serious and has the highest potential ecological risk, which is 4.44 times to 15.94 times higher than the local soil background value, causing serious environmental pollution and harm to agricultural production and ecosystems. In addition, agricultural products grown in these areas may also pose some potential health risks [38].

Soil pH values were weakly alkaline in the vegetable field (S2), which may be because local residents dump fly ash onto vegetable soil, thereby increasing the pH [9]. The abandoned land and the paddy fields on both sides of the river were acidic, probably because the acid ore water flowing down the mine flows into the river, causing the residents to use the river water when irrigating the farmland, thus having a certain impact on the pH of the soil. On the other hand, paddy fields have long been flooded by acid-containing mine wastewater, which seriously affects the soil properties and makes the soil pH weakly acidic [40].

Certain soil physicochemical properties have a positive impact on soil fertility levels and can increase soil porosity, transport, storage, and nutrient cycling [41]. In this study, TOC, TN, and AK in the paddy soil were significantly higher than those in the abandoned land. It is possible that the vegetable field and the paddy field are the main crop production areas, and the nutrient source was better than that in the abandoned land. However, it is worth noting that the TP of the abandoned land was significantly higher than that in other areas. This may be due to a large amount of phosphorus in the wastewater produced during the beneficiation of the ore dressing area, resulting in a large amount of P in the abandoned land nearby. Pearson correlations indicated (Table S3) that Cu, Zn, Pb, and Cd were significantly positively correlated with TP, which may be related to the partial heavy metals in phosphate fertilizer [39]. While heavy metals had a significant negative correlation with TOC, it is possible that the organic component has a ligand or group that can form a chelate with the metal, thereby resulting in a high affinity for heavy metal cations, such as Cu, Cd, and Pb [42].

### 4.2. Effects of Heavy Metal Pollution on Soil Microbial Communities

This study found that the soil environment at different distances on the two sides of the Shun’an River near the mining area was significantly different (Figure 2, Tables S1 and S2). In addition to being affected by varying degrees of heavy metal pollution, microbial communities adapt to new habitats by altering their abundance and structure [43]. This is similar to the previous research results, that is, how the microbial community structure in the soil varies with distance, plant type, and among habitats with different levels of heavy metal pollution [43,44]. Zhang et al. [35] found that heavy metals can induce the establishment of specific microbial communities. In this study, Chloroflexi, Verrucomicrobia, Nitrospirae, Patescibacteria, and Crenarchaeota had low levels of abundance or were not abundant in the areas that were heavily contaminated by heavy metals (i.e., S1, S2, and S5) closest to the mine (Figure 4). This indicates that these bacteria are sensitive to heavy metals and cannot adapt to such heavily polluted living environments. In contrast, Proteobacteria, Bacteroidetes, Actinobacteria, and Thaumarchaeota were more abundant in areas with less pollution than the mining area, and the correlation heatmap (Figure 5) showed the same results. These results indicate that these microorganisms can well-tolerate heavy metals and can adapt to survival and reproduction in this environment. Some studies have found that these bacteria survive under heavy metal stress because they have a chelating ability, allowing them to be resistant to heavy metals [42]. In addition, previous studies have shown that gram-negative bacteria are more extensive than gram-positive bacteria in places where heavy metals occur, because the cell walls of gram-positive bacteria bind better to metal cations [45].

The two most dominant bacteria in the soil samples under different plant types on the banks of the Shun’an River around the mining area were Proteobacteria and Acidobacteria, accounting for half of the total microbial community. This may be due to the heavy metal tolerance of the two strains, while sensitive microbes were less abundant [44]. For example, Proteobacteria species are often found in polluted soils and uncontaminated soils [16], and Proteobacteria is also a dominant phylum in many mine soils [9]. It has also been found in previous studies that Proteobacteria species are highly resistant to some heavy metals [14]. This study also had the same findings. On the one hand, Proteobacteria at the most polluted site, S1 was the most abundant, while the Proteobacteria in the remaining sampling areas increased with distance along the banks of the Shun’an River. On the other hand, except for Zn and Ni, which showed no correlation with Proteobacteria, other heavy metals were positively correlated, indicating that Proteobacteria species are resistant to the heavy metals Cu, Pb, and Cd. At the class level, Gammaproteobacteria is the most dominant bacteria and is positively correlated with both heavy metals and *P_N_* (Figure S6). This indicates that Gammaproteobacteria is strongly tolerant, which is similar to the results found by Yin et al. [46] for river sediments but contrary to the results obtained by Gillan et al. [47] for marine sediments. The Deltaproteobacteria and Alphaproteobacteria in this study showed a negative correlation with heavy metals and their combined pollution index. However, contrary to the study of Sandaa et al. [48], Alphaproteobacteria in heavy metal-contaminated soils increased with increasing pollution. Differences in the above-mentioned research results may also involve the role of other environmental factors. Bouskill et al. [49] explains this adaptation mechanism as Proteobacteria’s complex lifestyle and its ability to degrade many complex organic molecules that make it adaptable to many environments.

Another dominant bacteria in this study is Acidobacteria, reported to be a dominant microbe in a variety of environments, such as mountain forests [50], farmland [51], and e-waste stations [52]. This study shows that Zn, Pb, Cd, and *P_N_* were positively correlated with Acidobacteria; a significant correlation was observed for Zn (Figure 5). At the same time, the community abundance at S2 and S5 near the mine was the highest (Figure 4a), which indicates that Acidobacteria is highly resistant to heavy metal contaminants.

In all the samples, the diversity of archaea was very low, and heavy metals had different effects on the structure and abundance of different archaea. The results of this study indicate that the differences in the richness and diversity of archaea were significant in different habitats. Thaumarchaeota was the most dominant archaea, accounting for 55.78% of all archaea, mainly distributed in areas with the most serious heavy metal pollution closest to the mine (S1, S2, S5). However, in the paddy soils near the mine (S3, S4, S6, and S7), Crenarchaeota was found to predominate in these areas, a result similar to that found by Wang et al. [18] for paddy fields irrigated by acid mine water. Sullivan et al. [53] also found that although the abundance of the archaea community in paddy soil varied with season and long-term fertilization, Crenarchaeota was always the dominant bacteria in the study area. In addition, Crenarchaeota was negatively correlated with most heavy metals and *P_N_* (Figure 6), which is consistent with the results of Crenarchaeota in heavily polluted areas (S1, S2, S5) in Figure 4b. In contrast, Cu pollution was not serious in paddy fields with a high nutrient content, which may be due to the tolerance of Crenarchaeota to a certain concentration of heavy metals or its preference to survive in regions with high moisture content and rich nutrients. Lam et al. [54] and Céline et al. [55] further studied the above conclusions and found that Crenarchaeota plays an important role in the nitrogen and carbon cycle in soil.

### 4.3. Correlation between Microbial Community Structure and Environmental Factors

It was found that the soil microbial communities in these seven sampling areas were roughly clustered into two groups (Figure 3, Figures S1–S3), which may result from the clustering of similar vegetation. Different vegetation may lead to different soil physical and chemical properties that then affect the survival of microbial communities; different patterns and properties include vegetable fields and paddy fields, as well as MC, pH, heavy metal content, nutrition, and other significant differences. This study found that MC, pH, and *P_N_* were the most important factors affecting the microbial community and structure in the region (Figure 6b). The corresponding conclusions from previous studies have also shown that the microbial community in the soil is formed by the combined effects of environmental factors and heavy metal pollution in the soil [56,57].

Bastida et al. [58] stated that soil irrigation production activities are another factor affecting the biomass, activity, and composition of soil microbial communities. Water is a key factor in maintaining soil microbial communities and affecting community composition and activity [59]. Consistent with our hypothesis, MC was found to be highly correlated with the functional structure of the soil microbial community and its distribution. Some studies have found that a certain reduction in MC can increase the abundance of microbial communities [60], but some studies have found that drought has a certain impact on microbial community composition [61]. In this study (Figure 5), Chloroflexi, Nitrospirae, and Crenarchaeota had a significant positive correlation with MC, and some studies found that the existence of these three bacteria are closely related to MC. Chloroflexi is often detected in seafloor sediments [62] and deep aquifers [63]. In contrast, Bacteroidetes and Thaumarchaeota were negatively correlated with the soil moisture content. Previous studies have shown that Bacteroidetes species are facultative aerobic bacteria, and in this study, Bacteroidetes and Thaumarchaeota may reflect aerobic conditions. Verrucomicrobia is generally considered to be one of the less common bacteria, which may be due to its nondominant abundance. However, many studies have found that Verrucomicrobia is almost ubiquitous in soil [64], and Bergmann et al. [65] found that Verrucomicrobia was most abundant in grassland and the 10–50 cm soil layer. In our study, Verrucomicrobia was the third most dominant bacterium, and its microbial abundance in the paddy field was higher than that in the vegetable and the abandoned land. This may be because Verrucomicrobia has a methane oxidation function; in other words, it can also survive through denitrification in the anoxic state [66].

Soil pH is related to soil microbial communities [67], especially bacterial communities, and the effect of pH on soil microorganisms is very complex [68]. Shen et al. [69] found that the relative abundance of soil bacteria was significantly positively correlated with pH, and the effects of other environmental factors (heavy metals and nutrients) on the bacterial community were also controlled by pH. The above results are similar to this study, and we also found that microbial communities had significant differences under different pH effects. This may be because pH affects the physiological and biochemical properties of microbial cells, thereby affecting the survival and growth of specific bacteria, ultimately altering the microbial community structure in the area [70]. On the other hand, soil pH can seriously affect the bioavailability of heavy metals and the activity of bacteria. Therefore, when selecting resistant bacteria for bioremediation of heavy metals in soil, the optimum pH value of soil should not be ignored. Our study found that Bacteroidetes had higher abundance in neutral and alkaline soils than in acidic soils, similar to the studies by Ganzert et al. [71] and Wolińska et al. [72]. The relative abundance of Acidobacteria increased with the increase in soil pH, which is different from the conclusion that Acidobacteria was negatively correlated with soil pH in the studies conducted by Liu et al. [73] and Jones et al. [74]. This may be because the pH of the soil in this study was between 6 and 8, and there was no significant acidity or alkalinity.

Nutrients are the basis for soil microbial survival. Some studies have shown that nutrients have a certain regulatory effect on the toxicity of heavy metals in soil [75,76], and fertilization can significantly change the composition of the soil community structure in agricultural systems [75,76]. In addition, Darcy and Schmidt [77] found that the application of nitrogen and phosphorus compound fertilizer significantly increased the abundance of Acidobacteria and promoted plant growth. This is consistent with the results of this study: Acidobacteria was the second most dominant bacteria and was positively correlated with phosphorus. However, fertilizers have different effects on microorganisms. Geisseler et al. [78] found that chemical fertilizer application had a positive or negative effect on the soil microbial biomass and activity and needed to be analyzed separately. In this study, the relationship between TN and TP on the microbial communities was completely opposite (Figure 5), indicating that the effects of different nutrients on microorganisms differ to some extent.

## 5. Conclusions

Based on 16S rRNA sequencing, this study provides novel and detailed insights into the microbial community composition and diversity under different heavy metal contamination levels and vegetation on both sides of the Shun’an River. Pollution distribution of heavy metals in soils was related with the distance from the mine. Microbial community abundance, structure, and soil habitat were significantly different depending on the contamination levels and planting types. The soil microbial communities were mainly affected by pH, heavy metal pollution, and vegetation. pH significantly affected the abundance and structure of most microorganisms. The dominant bacteria Proteobacteria, Bacteroidetes, Actinobacteria, and Thaumarchaeota are highly tolerant to heavy metals and can be used in future soil-testing experiments and to improve soil conditions. Our results offer a better understanding of the crucial factors on microbial community structure at heavy metal contamination associated with vegetation in the vicinity of the mine.

## Supplementary Materials

The following are available online at https://www.mdpi.com/1660-4601/17/16/5680/s1, Table S1: Mine soil physical and chemical properties and evaluation of heavy metal pollution, Table S2: Microbial community diversity index (97% sequence similarity), Table S3: Pearson correlation analysis between heavy metals and environmental factors, Figure S1: Microbial community dilution curve, Figure S2: Venn diagram of sampling points S1, S2, S5, Figure S3: Venn diagram of sampling points S3, S4, S6, and S7, Figure S4: Similarity metric heat map based on the Bray-Curtis distance, Figure S5: Relative abundance (%) of dominant bacteria at the class level, Figure S6: Correlation between soil physical and chemical properties and heavy metals and microbial community abundance.

## References

[1] Kicińska A. (2019). Environmental risk related to presence and mobility of As, Cd and Tl in soils in the vicinity of a metallurgical plant—Long-term observations. Chemosphere.

[2] Kicińska A., Gruszecka-Kosowska A. (2016). Long-term changes of metal contents in two metallophyte species (Olkusz area of Zn-Pb ores, Poland). Environ. Monit. Assess..

[3] Li Z., Ma Z., van der Kuijp T.J., Yuan Z., Huang L. (2014). A review of soil heavy metal pollution from mines in China: Pollution and health risk assessment. Sci. Total Environ..

[4] Bernhardt E.S., Lutz B.D., King R.S., Fay J.P., Carter C.E., Helton A.M., Campagna D., Amos J. (2012). How many mountains can we mine? Assessing the regional degradation of Central Appalachian rivers by surface coal mining. Environ. Sci. Technol..

[5] Huang Y., Chen Q., Deng M., Japenga J., Li T., Yang X., He Z. (2018). Heavy metal pollution and health risk assessment of agricultural soils in a typical peri-urban area in southeast China. J. Environ. Manage..

[6] Lu Y., Song S., Wang R., Liu Z., Meng J., Sweetman A.J., Jenkins A., Ferrier R.C., Li H., Luo W. (2015). Impacts of soil and water pollution on food safety and health risks in China. Environ. Int..

[7] Chodak M., Gołebiewski M., Morawska-Płoskonka J., Kuduk K., Niklińska M. (2013). Diversity of microorganisms from forest soils differently polluted with heavy metals. Appl. Soil Ecol..

[8] Vinhal-Freitas I.C., Corrêa G.F., Wendling B., Bobuľská L., Ferreira A.S. (2017). Soil textural class plays a major role in evaluating the effects of land use on soil quality indicators. Ecol. Indic..

[9] Zhao X., Huang J., Lu J., Sun Y. (2019). Study on the influence of soil microbial community on the long-term heavy metal pollution of different land use types and depth layers in mine. Ecotoxicol. Environ. Saf..

[10] Tischer A., Blagodatskaya E., Hamer U. (2015). Microbial community structure and resource availability drive the catalytic efficiency of soil enzymes under land-use change conditions. Soil Biol. Biochem..

[11] Teklay T., Shi Z., Attaeian B., Chang S.X. (2010). Temperature and substrate effects on C & N mineralization and microbial community function of soils from a hybrid poplar chronosequence. Appl. Soil Ecol..

[12] Balogh J., Pintér K., Fóti S., Cserhalmi D., Papp M., Nagy Z. (2011). Dependence of soil respiration on soil moisture, clay content, soil organic matter, and CO2 uptake in dry grasslands. Soil Biol. Biochem..

[13] Jiang B., Adebayo A., Jia J., Xing Y., Deng S., Guo L., Liang Y., Zhang D. (2019). Impacts of heavy metals and soil properties at a Nigerian e-waste site on soil microbial community. J. Hazard. Mater..

[14] Zhang J., Wang L.H., Yang J.C., Liu H., Dai J.L. (2015). Health risk to residents and stimulation to inherent bacteria of various heavy metals in soil. Sci. Total Environ..

[15] Azarbad H., Niklińska M., Laskowski R., van Straalen N.M., van Gestel C.A.M., Zhou J., He Z., Wen C., Röling W.F.M. (2015). Microbial community composition and functions are resilient to metal pollution along two forest soil gradients. FEMS Microbiol. Ecol..

[16] Pereira L.B., Vicentini R., Ottoboni L.M. (2014). Changes in the bacterial community of soil from a neutral mine drainage channel. PLoS ONE.

[17] Hu X.F., Jiang Y., Shu Y., Hu X., Liu L., Luo F. (2014). Effects of mining wastewater discharges on heavy metal pollution and soil enzyme activity of the paddy fields. J. Geochemical Explor..

[18] Wang H., Zeng Y., Guo C., Bao Y., Lu G., Reinfelder J.R., Dang Z. (2018). Bacterial, archaeal, and fungal community responses to acid mine drainage-laden pollution in a rice paddy soil ecosystem. Sci. Total Environ..

[19] DeNicola D.M., Stapleton M.G. (2014). Benthic diatoms as indicators of long-term changes in a watershed receiving passive treatment for acid mine drainage. Hydrobiologia.

[20] Beattie R.E., Henke W., Campa M.F., Hazen T.C., McAliley L.R., Campbell J.H. (2018). Variation in microbial community structure correlates with heavy-metal contamination in soils decades after mining ceased. Soil Biol. Biochem..

[21] Fan M., Lin Y., Huo H., Liu Y., Zhao L., Wang E., Chen W., Wei G. (2016). Microbial communities in riparian soils of a settling pond for mine drainage treatment. Water Res..

[22] Sun Z., Xie X., Wang P., Hu Y., Cheng H. (2018). Heavy metal pollution caused by small-scale metal ore mining activities: A case study from a polymetallic mine in South China. Sci. Total Environ..

[23] Xiao R., Wang S., Li R., Wang J.J., Zhang Z. (2017). Soil heavy metal contamination and health risks associated with artisanal gold mining in Tongguan, Shaanxi, China. Ecotoxicol. Environ. Saf..

[24] Asakawa S., Kimura M. (2008). Comparison of bacterial community structures at main habitats in paddy field ecosystem based on DGGE analysis. Soil Biol. Biochem..

[25] Wang S.H., Yang J., Liu S.M., Zhao-Wen X.U., Xian-Cai L.U., Wang B., Wang H. (2011). Environmental Effects of Heavy Metal Elements Release in Yangshanchong Tailing Pool, Shizishan, Tongling, Anhui Province. Geol. J. China Univ..

[26] Fu H., Ma Y., Wu Y., Hu H., Wang Q., Ma T., Xu L., Nie J., Cheng W., He X. (2014). Study on the status of heavy metal pollution in Tongling mining area and farmland soil. J. Agric..

[27] Rodríguez L., Ruiz E., Alonso-Azcárate J., Rincón J. (2009). Heavy metal distribution and chemical speciation in tailings and soils around a Pb-Zn mine in Spain. J. Environ. Manage..

[28] Lv H., Liang Z. (2012). Dynamics of soil organic carbon and dissolved organic carbon in robina pseudoacacia forests. J. Soil Sci. Plant Nutr..

[29] Pan P., Kang Q., Li X. (2003). Determination of Total Phosphorus in Soil by Ammonium Molybdate Spectrophotometry. Chin. J. Spectrosc. Labortary.

[30] Guan Y., Shao C., Ju M. (2014). Heavy metal contamination assessment and partition for industrial and mining gathering areas. Int. J. Environ. Res. Public Health.

[31] Hakanson L. (1980). An ecological risk index for aquatic pollution control.a sedimentological approach. Water Res..

[32] Turnbaugh P.J., Hamady M., Yatsunenko T., Cantarel B.L., Duncan A., Ley R.E., Sogin M.L., Jones W.J., Roe B.A., Affourtit J.P. (2009). A core gut microbiome in obese and lean twins. Nature.

[33] Edgar R.C. (2010). Search and clustering orders of magnitude faster than BLAST. Bioinformatics.

[34] Jiang Z.Y., Wang Y.S., Cheng H., Zhang J.D., Fei J. (2015). Spatial variation of phytoplankton community structure in Daya Bay, China. Ecotoxicology.

[35] Zhang W., Guo R., Yang Y., Ding J., Zhang Y. (2016). Long-term effect of heavy-metal pollution on diversity of gastrointestinal microbial community of Bufo raddei. Toxicol. Lett..

[36] Wang N., Wang A., Xie J., He M. (2019). Responses of soil fungal and archaeal communities to environmental factors in an ongoing antimony mine area. Sci. Total Environ..

[37] Li R., Xu J., Jiang Y., Chen J., Ding G. (2013). Fraction Distribution and Ecological Risk Assessment of Soil Heavy Metals in the Riparian Zone of Huixi Stream in Tongling City. Res. Environ. Sci..

[38] Yang Q., Li Z., Lu X., Duan Q., Huang L., Bi J. (2018). A review of soil heavy metal pollution from industrial and agricultural regions in China: Pollution and risk assessment. Sci. Total Environ..

[39] Seshadri B., Bolan N.S., Wijesekara H., Kunhikrishnan A., Thangarajan R., Qi F., Matheyarasu R., Rocco C., Mbene K., Naidu R. (2016). Phosphorus-cadmium interactions in paddy soils. Geoderma.

[40] Kim K.H., Ammons J.T., Lee S.Y. (1990). Immobilization of Radioactive Strontium in Contaminated Soils by Phosphate Treatment. Mrs Proc..

[41] Smebye A., Alling V., Vogt R.D., Gadmar T.C., Mulder J., Cornelissen G., Hale S.E. (2016). Biochar amendment to soil changes dissolved organic matter content and composition. Chemosphere.

[42] Bolan N.S., Choppala G., Kunhikrishnan A., Park J., Naidu R. (2013). Microbial transformation of trace elements in soils in relation to bioavailability and remediation. Rev. Environ. Contam. Toxicol..

[43] Jayanthi B., Emenike C.U., Agamuthu P., Simarani K., Mohamad S., Fauziah S.H. (2016). Selected microbial diversity of contaminated landfill soil of Peninsular Malaysia and the behavior towards heavy metal exposure. Catena.

[44] Singh B.K., Quince C., Macdonald C.A., Khachane A., Thomas N., Abu-Alsoud W., Sørensen S.J., He Z., White D., Sinclair A. (2014). Loss of microbial diversity in soils is coincident with reductions in some specialized functions. Environ. Microbiol..

[45] Çolak F., Atar N., Yazicioĝlu D., Olgun A. (2011). Biosorption of lead from aqueous solutions by Bacillus strains possessing heavy-metal resistance. Chem. Eng. J..

[46] Yin H., Niu J., Ren Y., Cong J., Zhang X., Fan F., Xiao Y., Zhang X., Deng J., Xie M. (2015). An integrated insight into the response of sedimentary microbial communities to heavy metal contamination. Sci. Rep..

[47] Gillan D.C., Danis B., Pernet P., Joly G., Dubois P. (2005). Structure of sediment-associated microbial communities along a heavy-metal contamination gradient in the marine environment. Appl. Environ. Microbiol..

[48] Sandaa R.A., Torsvik V. (2001). Enger Influence of long-term heavy-metal contamination on microbial communities in soil. Soil Biol. Biochem..

[49] Bouskill N.J., Barker-Finkel J., Galloway T.S., Handy R.D., Ford T.E. (2010). Temporal bacterial diversity associated with metal-contaminated river sediments. Ecotoxicology.

[50] Meng H., Li K., Nie M., Wan J.R., Quan Z.X., Fang C.M., Chen J.K., Gu J.D., Li B. (2013). Responses of bacterial and fungal communities to an elevation gradient in a subtropical montane forest of China. Appl. Microbiol. Biotechnol..

[51] Eichorst S.A., Kuske C.R., Schmidt T.M. (2011). Influence of plant polymers on the distribution and cultivation of bacteria in the phylum acidobacteria. Appl. Environ. Microbiol..

[52] Wu W., Dong C., Wu J., Liu X., Wu Y., Chen X., Yu S. (2017). Ecological effects of soil properties and metal concentrations on the composition and diversity of microbial communities associated with land use patterns in an electronic waste recycling region. Sci. Total Environ..

[53] Sullivan T.S., McBride M.B., Thies J.E. (2013). Soil bacterial and archaeal community composition reflects high spatial heterogeneity of pH, bioavailable Zn, and Cu in a metalliferous peat soil. Soil Biol. Biochem..

[54] Lam P., Jensen M.M., Lavik G., McGinnis D.F., Müller B., Schubert C.J., Amann R., Thamdrup B., Kuypers M.M.M. (2007). Linking crenarchaeal and bacterial nitrification to anammox in the Black Sea. Proc. Natl. Acad. Sci. USA.

[55] Céline B.-A., Boussau B., Simonetta G., Patrick F. (2008). Mesophilic crenarchaeota: Proposal for a third archaeal phylum, the Thaumarchaeota. Nat. Rev. Microbiol..

[56] Zhao Q., Li R., Ji M., Ren Z. (2016). Organic content influences sediment microbial fuel cell performance and community structure. Bioresour. Technol..

[57] Frossard A., Hartmann M., Frey B. (2017). Tolerance of the forest soil microbiome to increasing mercury concentrations. Soil Biol. Biochem..

[58] Bastida F., Torres I.F., Andrés-Abellán M., Baldrian P., López-Mondéjar R., Větrovský T., Richnow H.H., Starke R., Ondoño S., García C. (2017). Differential sensitivity of total and active soil microbial communities to drought and forest management. Glob. Chang. Biol..

[59] Hackl E., Pfeffer M., Donat C., Bachmann G., Zechmeister-Boltenstern S. (2005). Composition of the microbial communities in the mineral soil under different types of natural forest. Soil Biol. Biochem..

[60] Barnard R.L., Osborne C.A., Firestone M.K. (2015). Changing precipitation pattern alters soil microbial community response to wet-up under a Mediterranean-type climate. ISME J..

[61] Canarini A., Kiær L.P., Dijkstra F.A. (2017). Soil carbon loss regulated by drought intensity and available substrate: A meta-analysis. Soil Biol. Biochem..

[62] Nakahara N., Takaki Y., Ogawara M., Yamaguchi T., Takai K., Imachi H. (2019). Complete genome sequence of Pelolinea submarina MO-CFX1T within the phylum Chloroflexi, isolated from subseafloor sediment. Mar. Genomics.

[63] Grégoire P., Fardeau M.L., Joseph M., Guasco S., Hamaide F., Biasutti S., Michotey V., Bonin P., Ollivier B. (2011). Isolation and characterization of Thermanaerothrix daxensis gen. nov., sp. nov., a thermophilic anaerobic bacterium pertaining to the phylum “Chloroflexi”, isolated from a deep hot aquifer in the Aquitaine Basin. Syst. Appl. Microbiol..

[64] Li Z., Xu Z. (2008). Assessing bacterial diversity in soil. J. Soils Sediments.

[65] Bergmann G.T., Bates S.T., Eilers K.G., Lauber C.L., Caporaso J.G., Walters W.A., Knight R., Fierer N. (2011). The under-recognized dominance of Verrucomicrobia in soil bacterial communities. Soil Biol. Biochem..

[66] Ojala D.S., Beck D.A.C., Kalyuzhnaya M.G. (2011). Genetic systems for moderately halo (alkali) philic bacteria of the genus Methylomicrobium. Method. Enzymol..

[67] Rousk J., Brookes P.C., Bååth E. (2009). Contrasting soil pH effects on fungal and bacterial growth suggest functional redundancy in carbon mineralization. Appl. Environ. Microbiol..

[68] Pietri J.C.A., Brookes P.C. (2008). Relationships between soil pH and microbial properties in a UK arable soil. Soil Biol. Biochem..

[69] Shen J.P., Zhang L.M., Guo J.F., Ray J.L., He J.Z. (2010). Impact of long-term fertilization practices on the abundance and composition of soil bacterial communities in Northeast China. Appl. Soil Ecol..

[70] Lauber C.L., Hamady M., Knight R., Fierer N. (2009). Pyrosequencing-based assessment of soil pH as a predictor of soil bacterial community structure at the continental scale. Appl. Environ. Microbiol..

[71] Ganzert L., Bajerski F., Wagner D. (2014). Bacterial community composition and diversity of five different permafrost-affected soils of Northeast Greenland. FEMS Microbiol. Ecol..

[72] Wolińska A., Kuźniar A., Zielenkiewicz U., Izak D., Szafranek-Nakonieczna A., Banach A., Błaszczyk M. (2017). Bacteroidetes as a sensitive biological indicator of agricultural soil usage revealed by a culture-independent approach. Appl. Soil Ecol..

[73] Liu C., Ding N., Fu Q., Brookes P.C., Xu J., Guo B., Lin Y., Li H., Li N. (2016). The influence of soil properties on the size and structure of bacterial and fungal communities along a paddy soil chronosequence. Eur. J. Soil Biol..

[74] Jones R.T., Robeson M.S., Lauber C.L., Hamady M., Knight R., Fierer N. (2009). A comprehensive survey of soil acidobacterial diversity using pyrosequencing and clone library analyses. ISME J..

[75] Eo J., Park K.-C. (2016). Long-term effects of imbalanced fertilization on the composition and diversity of soil bacterial community. Agric. Ecosyst. Environ..

[76] Yu C., Hu X.M., Deng W., Li Y., Xiong C., Ye C.H., Han G.M., Li X. (2015). Changes in soil microbial community structure and functional diversity in the rhizosphere surrounding mulberry subjected to long-term fertilization. Appl. Soil Ecol..

[77] Darcy J.L., Schmidt S.K. (2016). Nutrient limitation of microbial phototrophs on a debris-covered glacier. Soil Biol. Biochem..

[78] Geisseler D., Scow K.M. (2014). Long-term effects of mineral fertilizers on soil microorganisms—A review. Soil Biol. Biochem..

