# Supplementary material for: Ecological Effects of Heavy Metal Pollution on Soil Microbial Community Structure and Diversity on Both Sides of a River around a Mining Area
Source: Int J Environ Res Public Health. 2020 Aug 6;17(16):5680. doi: 10.3390/ijerph17165680 (PMC7460318; doi:10.3390/ijerph17165680)
Supplement: Supplementary file 1 [file ijerph-17-05680-s001.pdf]

Supporting Information

Ecological effects of heavy metal pollution on soil microbial community structure and diversity on both sides of a river around a mining area

International Journal of Environmental Research and Public Health

Xingqing Zhao[1,*], Jian Huang[1], Xuyan Zhu[1], Jinchun Chai[2] and Xiaoli Ji[3,*]

[1] School of Environmental and Safety Engineering, Changzhou University, Changzhou 213164, China;

[2] Department of Civil Engineering and Architecture, Saga University, Saga 8408502, Japan

[3] School of Economics, Changzhou University, Changzhou 213164, China;

* Correspondence: zhaoxq@cczu.edu.cn (X.Zhao)； xl.ji@163.com (X.J.)

Supplementary materials

Supplementary materials Table S1 Mine soil physical and chemical properties and evaluation of heavy metal pollution.

Sample area	pH	Cu (mg/kg)	Zn (mg/kg)	Pb (mg/kg)	Cd (mg/kg)	Ni (mg/kg)	P_N	RI	MC (g/kg)	TOC (g/kg)	TN (mg/kg)	TP (mg/kg)	AK (mg/kg)
S1	6.28± 0.19a	1274.5± 59.74e	594.31± 61.97c	616.94± 74.42c	11.16± 2.04c	46.06± 12.45ab	13.18± 1.01c	609.24± 54.01d	0.07± 0.02b	3.69± 0.64a	977.46± 69.1a	801.61± 56.87d	53.45± 14.19a
S2	7.79± 0.59b	240.57± 81.72b	314.34± 66.82b	193.23± 12.89b	6.7± 1.32ab	67.77± 0.84c	6.99± 0.58b	325.06± 32.2c	0.04± 0.01a	10.15± 1.1bc	1265.20± 135.01b	458.25± 16.05c	95.78± 18.81ab
S3	6.33± 0.05a	317.7± 28.73b	132.59± 28.4a	96.13± 8.45a	5.79± 0.6ab	50.8± 3.47b	6.20± 0.64b	280.47± 28.71	0.15± 0.02cd	16.12± 1.08d	1489.23± 109.43b	358.95± 57.4ab	110.3± 19.71bc
S4	6.11± 0.04a	397.13± 13.11c	276.31± 11.53b	70.48± 5.99a	5.02± 0.14ab	43.53± 3.87b	5.51± 0.38ab	251.04± 14.87ab	0.15± 0.02d	9.47± 0.73b	2455.69± 110.09d	485.17± 14.97c	168.8± 14.71c
S5	7.02± 0.66a	634.4± 36.15d	145.18± 13.7a	55.21± 6.04a	4.33± 0.46ab	37.45± 1.17ab	6.17± 0.48b	233± 15.56ab	0.09± 0.01bc	12.33± 0.26c	1418.08± 9.92b	428.52± 54.82c	151.1± 21.07c
S6	6.18± 0.07a	128.95± 11.39a	106.96± 6.33a	38.59± 4.53a	5.00± 0.16ab	44.45± 1.73b	5.24± 0.17ab	229.9± 8.43ab	0.11± 0.01bcd	8.90± 0.63b	1190.94± 94.79b	257.92± 18.33a	138± 9.9bc
S7	6.24± 0.12a	114.66± 8.23b	107.06± 18.12a	59.09± 6.12a	3.11± 0.29a	22.48± 6.22a	3.35± 0.31a	149.12± 16.1a	0.10± 0.01bcd	15.14± 0.73d	1776.22± 152.4c	400.03± 41.61bc	168.5± 25.85d

Note: The results are expressed as the mean ± standard error a;

a: Indicates significance difference among 7 samples in different areas;

Total organic carbon (TOC); Total nitrogen (TN); Total phosphorus (TP); Available potassium (AK); Moisture content (MC).

P_N: (Nemerow's pollution index).

The Grading Standards: $P_N \leq 0.7$, safety; $0.7 < P_N \leq 1$, warning limit; $1 < P_N \leq 2$, slight pollution; $2 < P_N \leq 3$, moderate pollution; $P_N > 3$, heavy pollution.

Potential ecological risk assessment (RI): RI <150, low potential ecological risk; 150 ≤ RI < 300, moderate ecological risk; 300 ≤ RI <600, considerable ecological risk; RI ≥ 600 very high ecological risk. Background values: (Cu:79.0 g/kg, Zn:139 g/kg, Pb:67 g/kg, Cd:0.71 g/kg, Ni:33 g/kg)

Supplementary materials Table S2 Microbial community diversity index (97% sequence similarity).

Sample area	No. of seqs		No. of OTUs		Chao1		Dominance		Observed species		Shannon		Simpson	
	Bacteria	Archaea	Bacteria	Archaea	Bacteria	Archaea	Bacteria	Archaea	Bacteria	Archaea	Bacteria	Archaea	Bacteria	Archaea
S1	39535	379	2852	26	3434.454	30.25173	0.006206	0.22032	2588	20	9.155878	1.624835	0.993794	0.779679
S2	30549	3933	1794	22	2109.61	23.69167	0.011783	0.250279	1770	16	8.488059	2.352446	0.988217	0.749721
S3	38080	1387	3236	106	3904.8	124.6617	0.005608	0.083883	2969	104	9.501584	4.644679	0.994392	0.916117
S4	36238	1592	3102	115	3824.674	135.7744	0.011628	0.069934	2885	107	9.277772	4.776795	0.988372	0.930066
S5	28925	5349	3001	53	3865.062	48.24848	0.003756	0.74109	3001	30	9.742548	1.07126	0.996244	0.25891
S6	39485	1281	3395	88	3941.841	102.8824	0.003816	0.133983	3105	88	9.805996	4.101433	0.996184	0.866017
S7	36483	1432	3414	101	3991.138	134.7559	0.003589	0.120121	3203	97	9.939062	4.162279	0.996411	0.879879

Supplementary materials Table S3 Pearson correlation analysis between heavy metals and environmental factors.

	pH	Cu	Zn	Pb	Cd	Ni	MC	TOC	TN	TP	AK	P_N	RI
pH	1												
Cu	-0.079	1											
Zn	0.076	0.829*	1										
Pb	-0.002	0.863*	0.942**	1									
Cd	0.046	0.818*	0.925**	0.955**	1								
Ni	0.610	0.052	0.356	0.243	0.470	1							
MC	-0.722	-0.269	-0.445	-0.471	-0.398	-0.337	1						
TOC	0.039	-0.675	-0.819*	-0.739	-0.769*	-0.284	0.412	1					
TN	-0.320	-0.370	-0.299	-0.518	-0.548	-0.321	0.637	0.343	1				
TP	-0.007	0.900**	0.945**	0.920**	0.828*	0.100	-0.381	-0.683	-0.210	1			
AK	-0.248	-0.647	-0.760*	-0.858*	-0.926**	-0.595	0.508	0.556	0.742	-0.643	1		
P_N	0.050	0.915**	0.918**	0.957**	0.977**	0.365	-0.409	-0.769*	-0.556	0.872**	-0.882**	1	
RI	0.027	0.874*	0.939**	0.971**	0.994**	0.396	-0.399	-0.772*	-0.529	0.874*	-0.900**	0.992**	1

Note: ** indicates an extremely significant correlation ($p < 0.01$), and * indicates a significant correlation ($p < 0.05$).

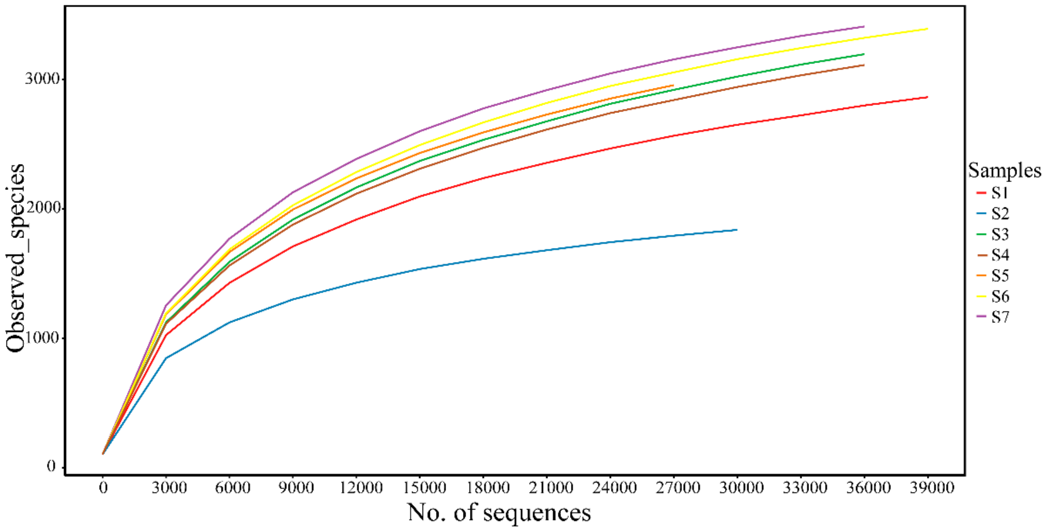

Supplementary materials Fig. S1 Microbial community dilution curve.

Note: Abandoned land (S1); Vegetable filed (S2, S5); Paddy field (S3, S4, S6, and S7).

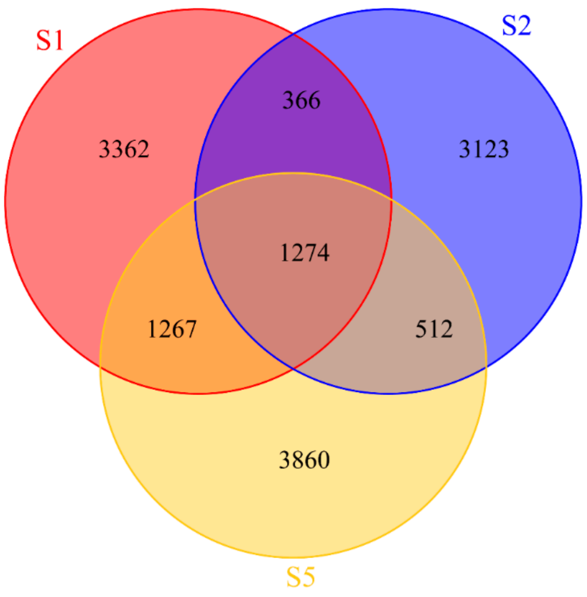

Supplementary materials Fig. S2 Venn diagram of sampling points S1, S2, S5.

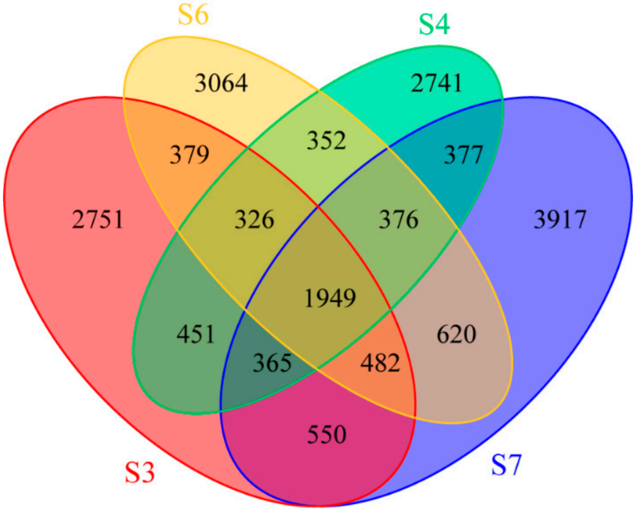

Supplementary materials Fig. S3 Venn diagram of sampling points S3, S4, S6, and S7.

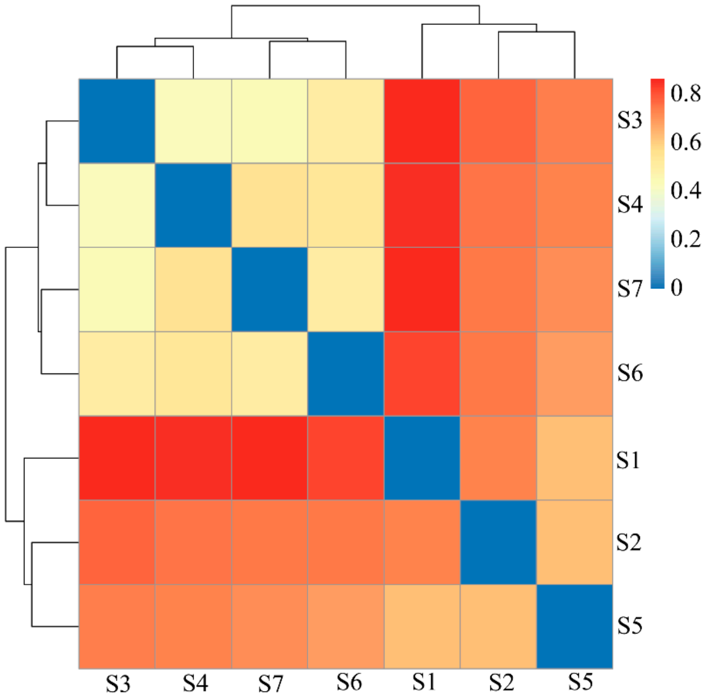

Supplementary materials Fig. S4 Similarity metric heat map based on the Bray-Curtis distance.

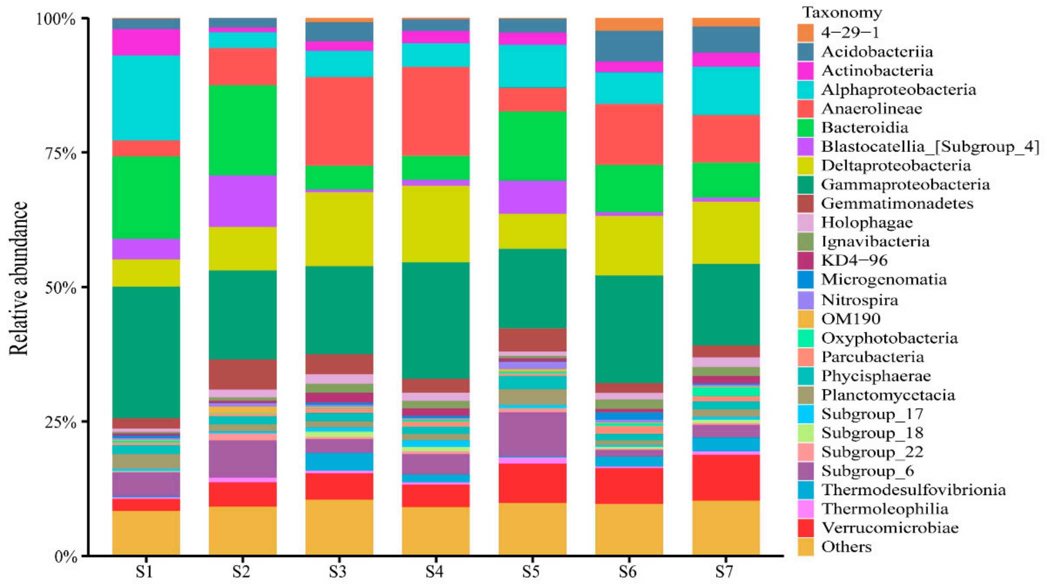

Supplementary materials Fig. S5 Relative abundance (%) of dominant bacteria at the class level.

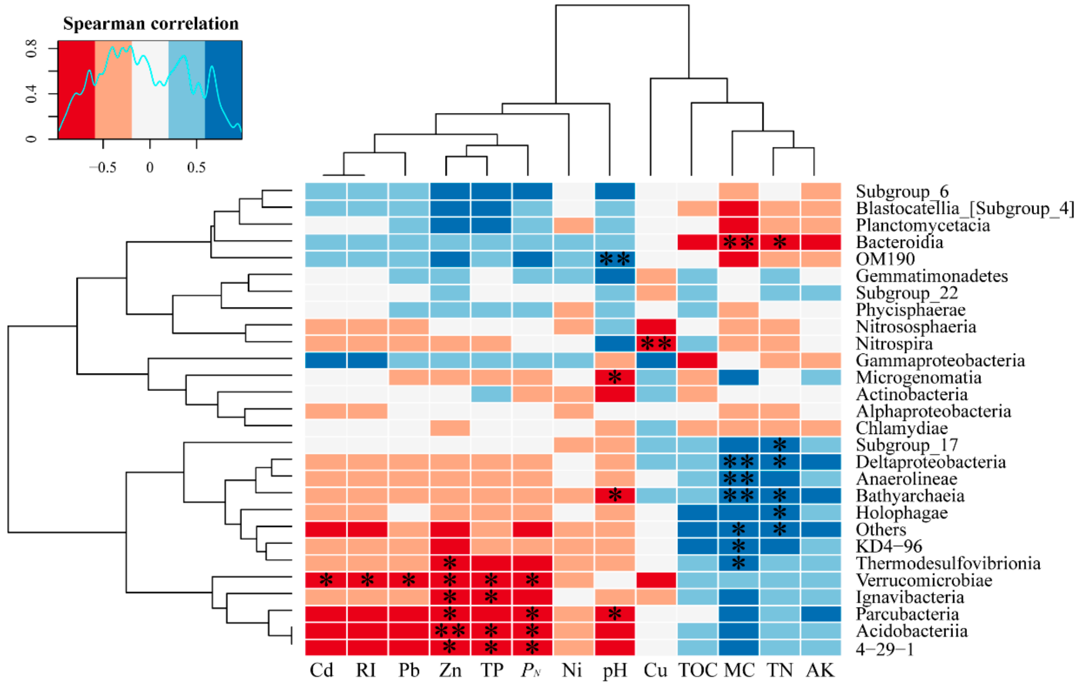

Supplementary materials Fig. S6 Correlation between soil physical and chemical properties and heavy metals and microbial community abundance.
